# Fenner’s Southern Medical Reports

**Published:** 1851-06

**Authors:** 


*Fenners' Southern Medical Reports.*— By a circular received some time since, and which should have received attention at our hands ere this, we are gratified to learn that Dr. Fenner's second volume of southern reports is in press, and will soon be issued at New-Orleans. Of the character of this enterprise we have already spoken. It is creditable alike to the country, to the contributions, and to the indefatigable editor. It is not, however, very creditable to the profession of this country, that, thus far, no adequate remuneration for the outlay of expense required for the publication of the work, has been received, putting out of the question the expenditure of time and labor. This effort in behalf of American medical literature, deserves more substantial evidences of appreciation than merely laudatory encouragement. Let all who can afford it become subscribers.

D. Davies, Son & Co., are the publishers, No. 57 Camp-street, New-Orleans.

